# COVID-19 Impact on Household Food Security in Urban and Peri-Urban Areas of Hyderabad, India

**DOI:** 10.3389/fpubh.2022.814112

**Published:** 2022-05-13

**Authors:** Ravula Padmaja, Swamikannu Nedumaran, Padmanabhan Jyosthnaa, Kasala Kavitha, Assem Abu Hatab, Carl-Johan Lagerkvist

**Affiliations:** ^1^Enabling Systems Transformation, International Crops Research Institute for the Semi-Arid Tropics, Patancheru, India; ^2^Department of Economics, Swedish University of Agricultural Sciences, Uppsala, Sweden; ^3^Department of Economics and Rural Development, Arish University, Arish, Egypt

**Keywords:** food security, pandemic (COVID-19), livelihood, coping strategies, urban, peri-urban, Hyderabad (India), India

## Abstract

This paper investigates the impact of the COVID-19 pandemic on food security and on coping-strategies in urban and peri-urban areas of the Hyderabad, India. Household survey data were collected before (October 2018) and during (January 2021) the onset of the pandemic. Results from logistic regression with the standarized Food Insecurity Expecience Scale (FIES) as dependent variable reveal that close to 40% of the households surveyed experienced a deterioration in food security status during the pandemic. In particular, we find that food security is closely related to the sector of employment in which the primary income- earning member of a household is engaged. To mitigate the impact of the pandemic on their food security, our sampled households adopted a variety of consumption-smoothing strategies including availing credit from both formal and informal sources, and liquidating their savings. Compared to households with severe or moderate level of food insecurity, households facing a mild level of food insecurity relied on stored food as a strategy to smoothen consumption in response to the income shock imparted by the pandemic. In addition, the results indicate that urban households, who adopted similar coping strategies as those adopted by peri-urban households, tended to be more food-insecure. Finally, the duration of unemployment experienced during the pandemic significantly influenced the status of household food security. These findings can inform the formulation of immediate and medium-term policy responses, including social protection policies conductive to mitigating the impacts of the COVID-19 pandemic and ameliorating the governance of urban food security during unexpected events and shocks.

## Introduction

On top of the direct health impacts of the COVID-19, the pandemic has disrupted food supply chains in developing countries, destabilized food prices and created profound negative effects on food security (1). In particular, the measures that governments in developing countries adopted to contain the spead of the virus have caused disruptions in transportation, manufacturing and service provisioning, which subsequently increased unemployment and caused an income loss estimated at USD 220 billion (2). These losses will reverberate across societies and impact education, human rights, and in most cases, basic food security and nutrition (3).

Economic lockdown and confinement measures implemented due to the pandemic have impacted employment across sectors within and between countries asymetrically (4–8). It has resulted in increased in unemployment rates, work from home arrangements and affected labor force participation. The overall economic downturn globally forced companies or firms to downsize their businesses, in some cases even complete shutdown, which got translated as reduced work hours for partial pay or losing their jobs entirely for many employees. The segments of the workforce most likely to be impacted are the most vulnerable groups, less educated low–wage workers, and those with non-standard contracts (temporary contracts, self-employed) (9, 10) and exacerbate the labor market iniquities.

In India, the sudden nationwide lockdown imposed by the national government from March 24, 2020 to May 31,2020 was one of the most extensive and stringent COVID-19 lockdowns in the world. In thus clamping down, the government's singular focus was on saving lives, not livelihoods. The lockdown froze economic activity across the country and delivered a large aggregate supply and demand shock to the economy. The consequences have been unprecedented in scale and intensity. Livelihoods were devastated due to the inability to maintain job security, food production was compromised and supply chains were distrupted. The adverse effects of a countrywide lockdown combined with weak political, economic, and social interventions had extended beyond income shocks and affected household food security (11, 12). Loss of employment, curtailed contracts and reduced wages exacerbated food insecurity risk (13).

These unfolding COVID-19 impacts on food security in developing countries have a strong territorial/spatial dimension (14), as regions have been heterogeneously affected in the short-run, and the medium- and long-term impact will vary significantly across regions. One of the greatest challenges facing the world's rapidly-growing urban population is how to access sufficient, affordable, and nutritious food (15). In particular, densely populated and deprived urban areas were the reportedly hardest hit than other areas (16). For many urban households, especially those living in poorer communities, labor is the most important asset. The fact that the majority of workers in such communities tend to work in the informal sector, earn a variable income and have little or no access to private or social insurance makes access to sufficient food a crucial issue (17). It is in this context that the global economic slowdown trigerred by the COVID-19 pandemic, as well as the disease itself, has exacerbated existing societal inequalities in most countries (7). Thus, COVID-19 impacts on food security in developing countries should be understood in the light of the rapid urbanization processes that many developing countries have been experiencing in recent decades. In this respect, evidence suggests that the burgeoning challenges posed by increased urbanization to the economic and social futures of developing countries through its effects on the resilience of food systems to unexpecteded shocks, such as disease outbreaks and other nature-induced changes. That is, urbanization is often associated with poverty, overburdening of social services, limited access to basic amenities and the resulting public health risks. The relationship between food security, food systems and sustainability needs to be given engaged consideration in the urban areas. Understanding this relationship is crucial because urban poverty and food insecurity are interrelated. However, there has been a lack of or limited systematic analysis of how urbanization affects contemporary food insecurity risk.

Literature on impacts of the pandemic on various sectors has been emerging since the onset of the global COVID 19 pandemic. Research has focused on impacts of the Covid 19 pandemic on social and associated psychological and health impacts due to the restrictions of social and physical mobility of people (18, 19) on the positive and negative environmental impacts (20–22), agriculture,supply chains and food systems (1, 23–26).

Extensive focus has been dedicated to observing the potential impacts of the pandemic on various economic indicators such as global poverty, government expenditures, budget deficits, employments etc. limited only to global and national scale (27–31). Contextualized data on the insidious growth and extensive impact of the COVID-19 pandemic on individuals and households (micro scale) is still emerging. The varying effects of COVID on the different economic strata needs to be assessed thoroughly on various economic parameters like livelihoods and income, access to markets etc. to build evidence that can support policy formation to develop robust coping strategies that ensure income smoothing and consumption.

Food security and financial security are fundamentally interconnected but there is sparse literature showing this connection. Income volatility has been gaining attention within the broader literature of economic well-being, and qualitative research suggests plausible association with the considerable challenges of meeting household food needs (32–35). Income shock and expenditure shock are strongly associated with food insecurity. Similarly, extended periods of unemployment increase the risks of food insecurity (36).

The combined effect of food price and income shocks arising from global crises such as the COVID-19 pandemic has been suggested as the likely cause of a sharp increase in hunger and poverty in low-income countries (37). There are reasons to expect that the pandemic has deeply altered food environments. First, the way people engage and interact with the food system to acquire, prepare and consume food has changed due to the lockdowns and the subsequent supply chain disruptions. Although most households in urban regions are net buyers of food, higher food prices are likely to have reduced household access to staple food. Secondly, the economy-wide negative impact of the pandemic and the subsequent lockdown which resulted in a loss of jobs across the country, has likely further limited households' ability to purchase food at higher prices (38). This only reinforces the need to understand the lockdown's impact on household food security status and coping mechanisms in the face of income shortfalls and food price shocks (39).

With this background, this study adds to the emerging literature on the impact of COVID-19 on food security in developing countries in several important ways. Leveraging on the multiple point data availability spanning across 4 years between 2018 and 2021 from a larger project the study makes a unique contribution to the emerging literature to understand the dynamics in the food security status in the aftermath of the pandemic and the phased lock down at a micro level using the food security status of the households prior to the pandemic in 2018 as benchmark capturing the spatial differences among urban and peri-urban households. We also attempt to assess the effect of pandemic on food security mediated through impact on changes in labour force participation and associated income loss.

In this paper, we seek to reach the following interrelated objectives:

- To analyze the impact of COVID-19 on the livelihoods of households residing in urban and peri-urban areas.
- To understand, by employing the Food Insecurity Experience Scale (FIES)^1^, the dynamics of food security at the household level in the context of the pandemic and the coping strategies employed to smoothen consumption.

## Materials and Methods

### Study Location

**Figure 1:**
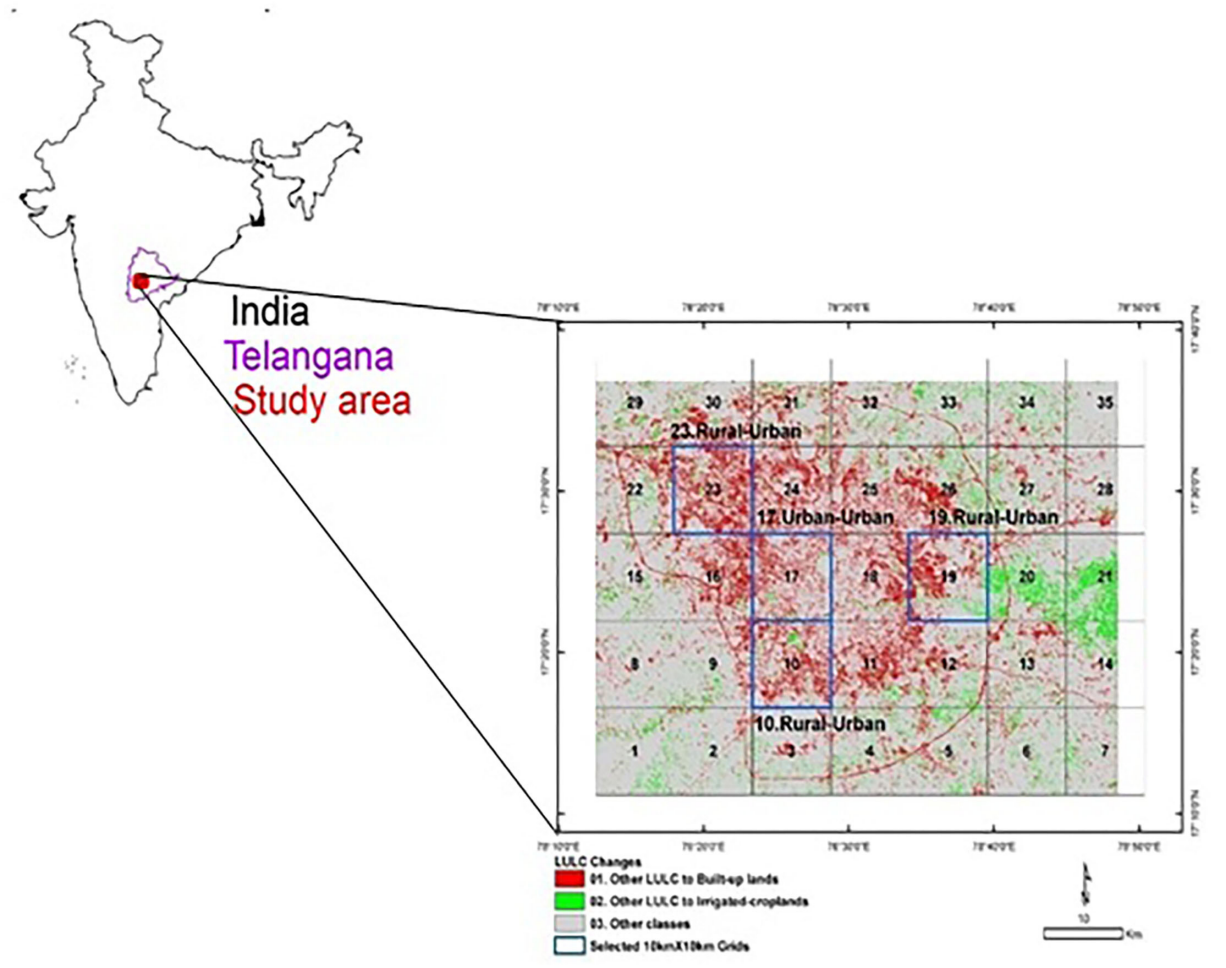
The grid of locations selected on the basis of GIS data for a study of the COVID-19 lockdown's impact on household food security in Hyderabad, India [based on Gumma et al. (40)].

The selection of the study area for the present study was based on the GIS/remote-sensing analysis by Gumma et al. (40), which assessed urban expansion and other land-use and land-cover changes in Hyderabad from 2005 to 2016. Using the outer ring road of Hyderabad as a boundary of the city (Figure 1) and following the method of Gumma et al. (40), we identified four quadrants/grids, each having similar features, on the map of Hyderabad: two grids in peri-urban areas and two in urban areas (Figure 1).

The population data of each mandal^2^ falling within the grid, fully or partly, were collected from the District Census Handbook 2011, and the proportion of geographic area contributed to the grid by each mandal was calculated. The mandal population in the respective grid area was proportional to its geographical area in the grid. The proportion of the geographical area of a mandal within the grid was multiplied by the total population of the mandal. Using this method, the total population of each grid and its contribution to the total sample were calculated (Table 1, Appendix 1). Refer to Supplementary Materials for appendices.

**Table 1:** Grid-wise proportionate sampling framework.

**Grid number**	**Population**	**Category**	**Sample proportion (%)**
10	209,524	Rural-Urban	16.51
17	347,141	Urban-Urban	27.36
19	230,543	Rural-Urban	18.17
23	461,156	Rural-Urban	36.34

### Household Selection

It should be highlighted that this study is part of a larger project in which a longitudinal panel of data was to be collected in four rounds (between 2018 to 2021) with the aim of identifying the status and implications of urbanization on food and nutrition security. The selection of the households was done in consultation with the local government workers (Anganwadi teachers^3^, sarpanches^4^, and ASHA^5^ workers) and based on the sampling strategy illustrated in the previous sub-section and presented in Table 1.

### Data Collection

Prior to data collection in the first round, a written approval for the survey was taken from the local administration of the Greater Hyderabad Municipal Corporation. The formal approval letters helped our personnel gain access to the chosen locations and elicit the cooperation of people in the community. As part of the ethical consideration, prior written consent was also taken from the respondents before each interview with the households.

The first round of data collection took place before the onset of the pandemic (October, 2018-February, 2019). In this round, 660 households were selected on the basis of the criteria laid down for this project as explained in the above section. The enumerators recorded the data on tablet computers using CsPro software^6^ For this present study, this round forms our baseline data against which we are measuring the changes. The second and third rounds of data collection for the project was carried out during June 2019 and November 2019, respectively. These rounds had some common modules from round one and also some additional modules. Data from these two rounds is not considered for this study.

After the onset of the COVID-19 pandemic in March 2020, from March 24 to May 31, 2020 strict lockdown restrictions were imposed nationwide in India on movement of goods and people. During the pandemic, there were three phases of withdrawal (unlock) of restrictions. The first unlock covered the period from June 1 to July 31, 2020 when certain essential services were restored and limited movement of people was allowed. The second unlock covered the period from August 1 to September 30, 2020 when there was a gradual opening up of the economy for a restricted time during the day and curfews were restricted to the late evening and night. The third unlock covered the period from October 1 to November 30, 2020, which saw the economy starting to get back to normalcy with restrictions on businesses and movement completely removed for all practical purposes.

During December 2020 to January 2021, a telephonic/remote survey was conducted on the same sample households as in the first round to understand the impact of COVID-19 pandemic on household food security. The telephonic survey covered the post-outbreak lockdown and three phases of withdrawal (unlock) of restrictions. The mobility restrictions due to the pandemic imposed by the Government of Telangana did not enable personal face to face interviews during this period. Out of the 660 households that were interviewed in round one, only 325 households could be interviewed through the telephonic survey. Audio recorded consent was taken from the households after the objectives of the survey were explained to the respondents. Data was recorded on tablet computers running KoBo Toolbox software^7^.

### Data and Variables Used in Analysis

The pre- and post-pandemic survey questionnaires including the standardized Food Insecurity Experience Scale (FIES) are presented in Appendix 2 and in Appendix 3, respectively. We would also like to particularly mention the following:

- The number of unemployed days for a household was calculated on the basis of the number of days of participation in the labor force during March—November 2020. The respondents self reported the availability of employment or non-employment during this period.
- The self-reported actual income and the approximate range of income received by the household during February 2020 was taken as the baseline to assess changes in income and income class. Income received in February 2020 served as the baseline as it was the closest proxy to liquid cash available within the household to meet immediate expenses during the lockdown.

We used 240 days as the benchmark figure for the purpose of our computation (of the number of unemployed days) as it was the maximum number of days that a primary income earner could have been employed across all types of employment^8^ during the above period. Thus, unemployment percentage was calculated from the number of days out of 240 that a household reported its primary earner as being out of employment. Households were categorized into three groups in terms of change in income status—“improved,” “reduced,” and “maintained status quo”—relative to the income range reported by them for the month of February 2020. The categorizing households by food insecurity level was done as per Ballard et al. (41). Household food insecurity was the outcome variable of interest in these models, whose covariates were categorical variables coded as 1 if the household was food-insecure, mildly food-insecure, or moderately food-insecure, and 0 if it was food-secure. The other categorical variables included in the model were area of residence, type of employment (and interaction between these two variables), coping strategies adopted, income, and number of unemployed days.

## Results

### Economic Loss

**Figure 2:**
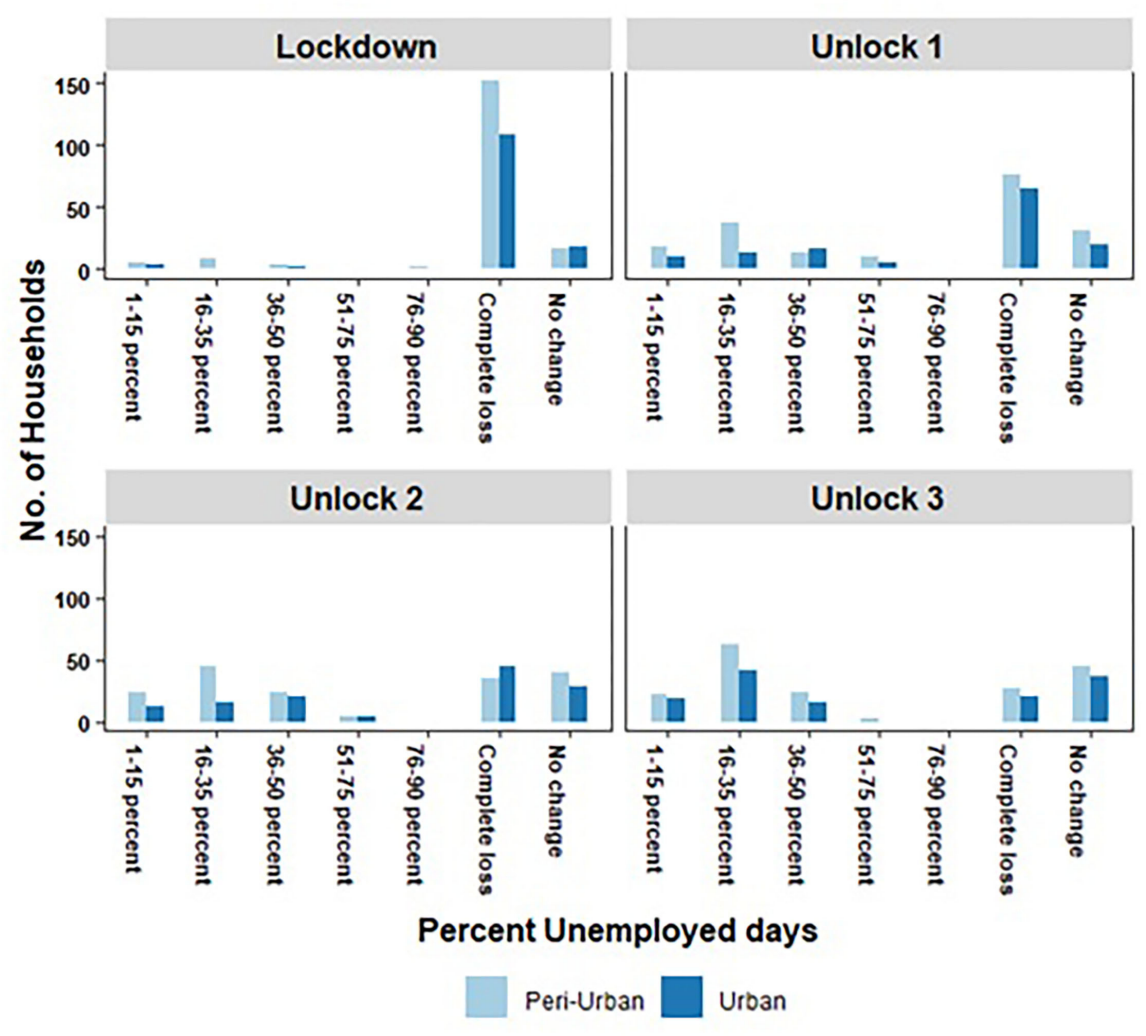
Unemployment (%) experienced by households in urban and peri-urban areas of Hyderabad, India during the COVID-19 lockdown and three-phased unlock (removal of restrictions).

From the information provided by each household on labor force participation by its primary income earner, we computed the number of unemployed days endured by the household during the lockdown and three phases of unlock, a period spanning from March 24 to November 30, 2020 (In our sample of households, we found that there was none that had more than one employed member at any time during this period.). During the lockdown period, the majority of households in our sample-except a small proportion-were not able to participate in the work force. In the subsequent three unlock phases, households located in the urban areas found it relatively easier to get back into the job market than peri-urban households. Accordingly, the percentage of unemployed days was relatively higher for peri-urban households compared to urban households (Figure 2).

**Figure 3:**
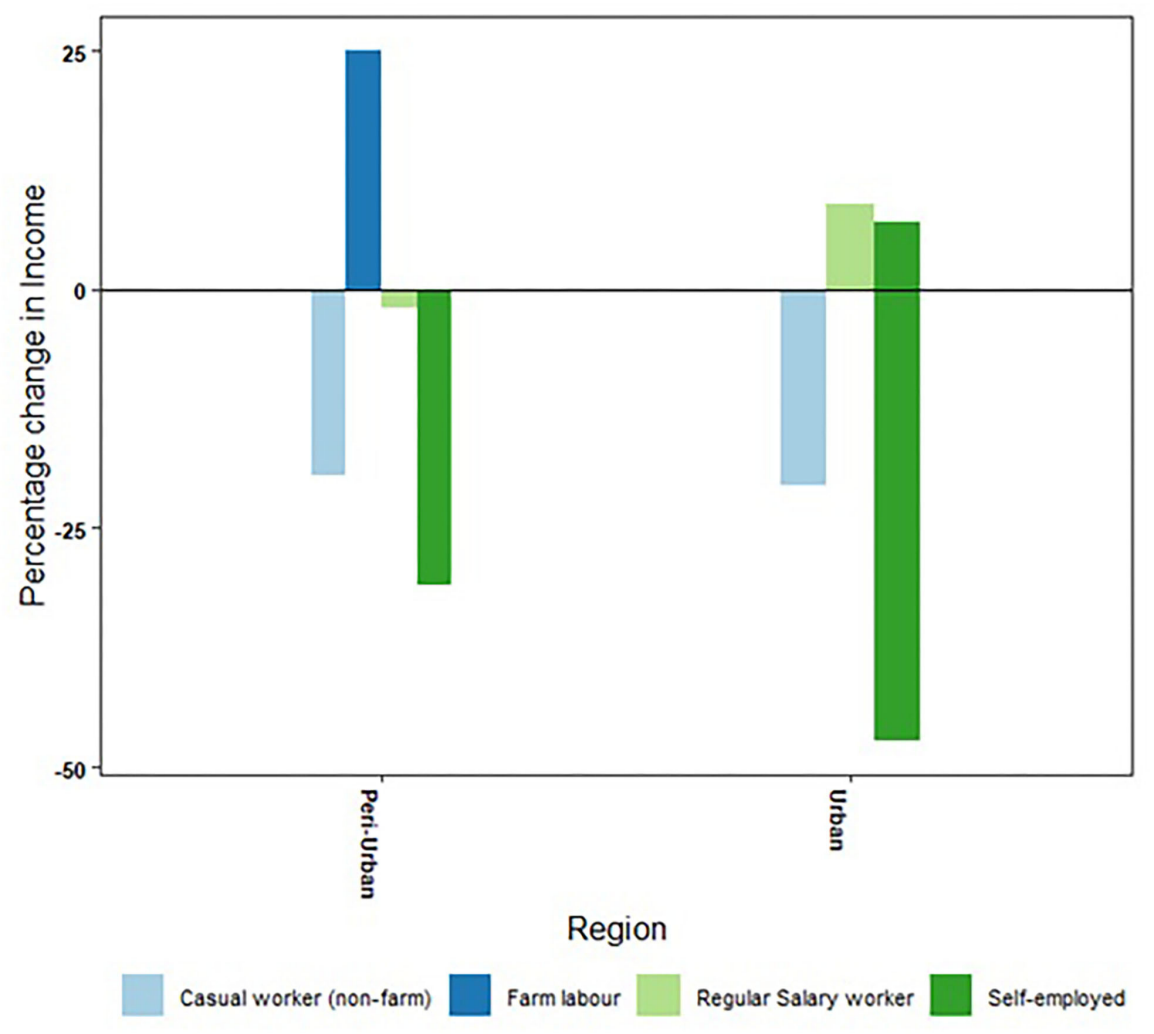
Change in income experienced by various categories of workers during the lockdown and unlock phases of the COVID-19 pandemic in urban and peri-urban areas of Hyderabad, India.

However, while urban households found it less difficult to get back to work, they experienced greater income reduction compared to their counterparts in the peri-urban areas. Income loss was particularly steeper for households engaged in a self-employed enterprise (with and without employees of their own). This could be attributed to the dampening of overall demand due to income and job losses as a consequence of the pandemic. The impact of dampening of overall demand is evident from the higher loss of income suffered by self-employed urban households with one or more employees compared to similar households in the peri-urban areas. Between urban and peri-urban areas, income losses sustained by other categories of households were comparable (Figure 3).

Analyzing the data for changes in household income status,^8^ we found that, in urban as well as peri-urban areas, the majority of households that depended on casual work, which was not related to agriculture or allied activities or were self-employed, experienced a reduction in income status. The number of such households reporting a lower income status during the pandemic was higher in the peri-urban areas. On the other hand, the majority of households that had a primary income earner in a regular salaried job did not experience a change in income status; however, 24% of such households did experience a reduction in income status, perhaps due to a pay cut. About 5% of households earning regular salaries improved their income status. These were households whose primary earners had jobs related to health sectors or had got into jobs offering a higher salary.

**Figure 4:**
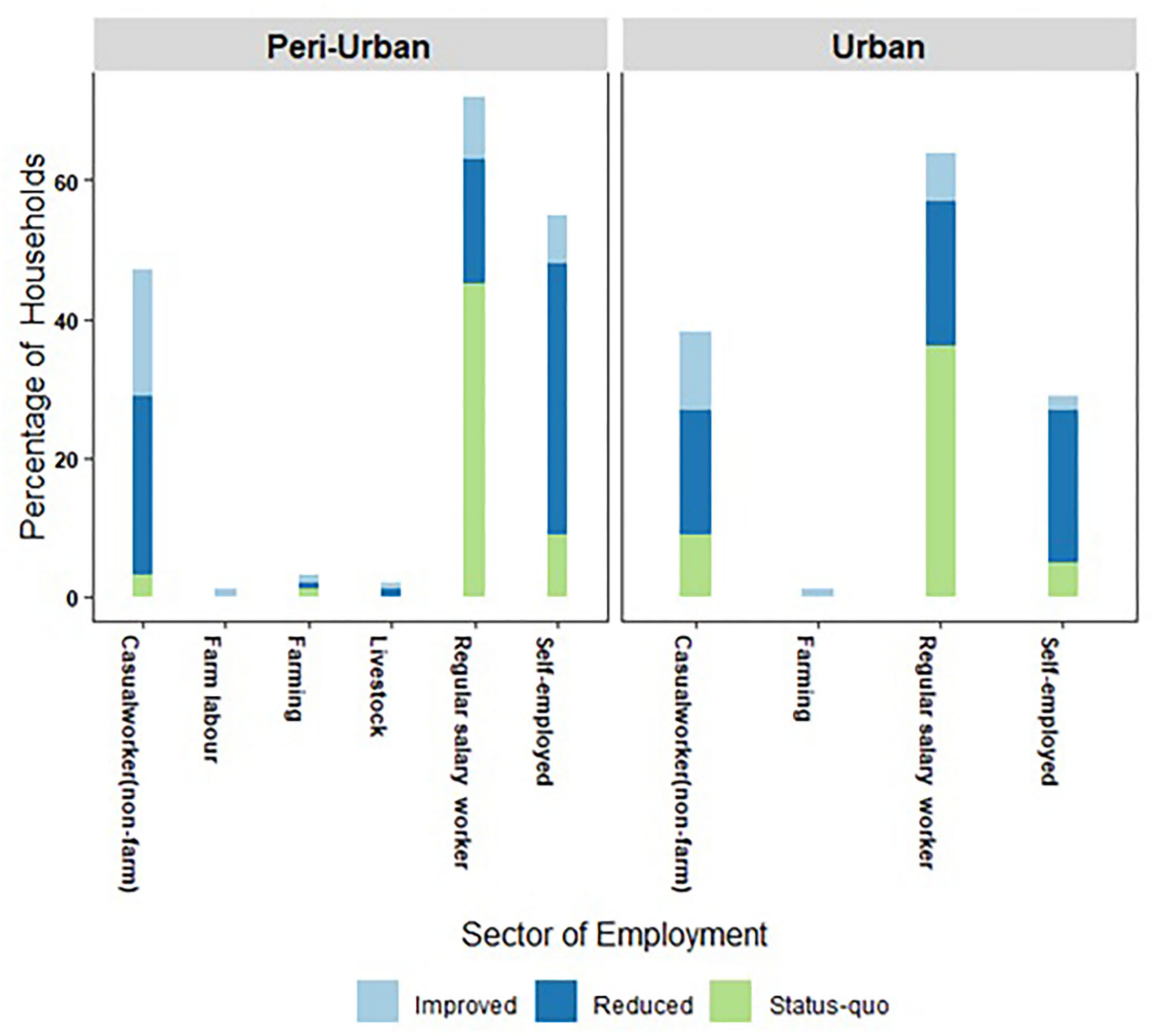
Change in household income status (improved/reduced/status quo) relative to pre-pandemic income levels experienced by different categories of households (categorized by type of employment) in urban and peri-urban areas of Hyderabad, India.

The pandemic and the restrictive measures taken by the government had a differential impact on different classes of workers; salaried workers having secure employment were the least affected in both urban and peri-urban areas. A comparison of the maximum incomes received by households during the lockdown and three phases of unlock with corresponding income data gathered in our pre-pandemic survey (October 2018 to February 2019) showed that most of the households that drew their income from regular salaried work with secure employment managed to maintain their income level or even saw a slight increase. In both urban and peri-urban areas, households whose income came from casual work in non-farm employment experienced a decline in income compared to the pre-lockdown period. The number of such households was slightly higher in the peri-urban areas (Figure 4).

### Food Insecurity

#### Background Characteristics of Households by Food Insecurity State

**Figure 5:**
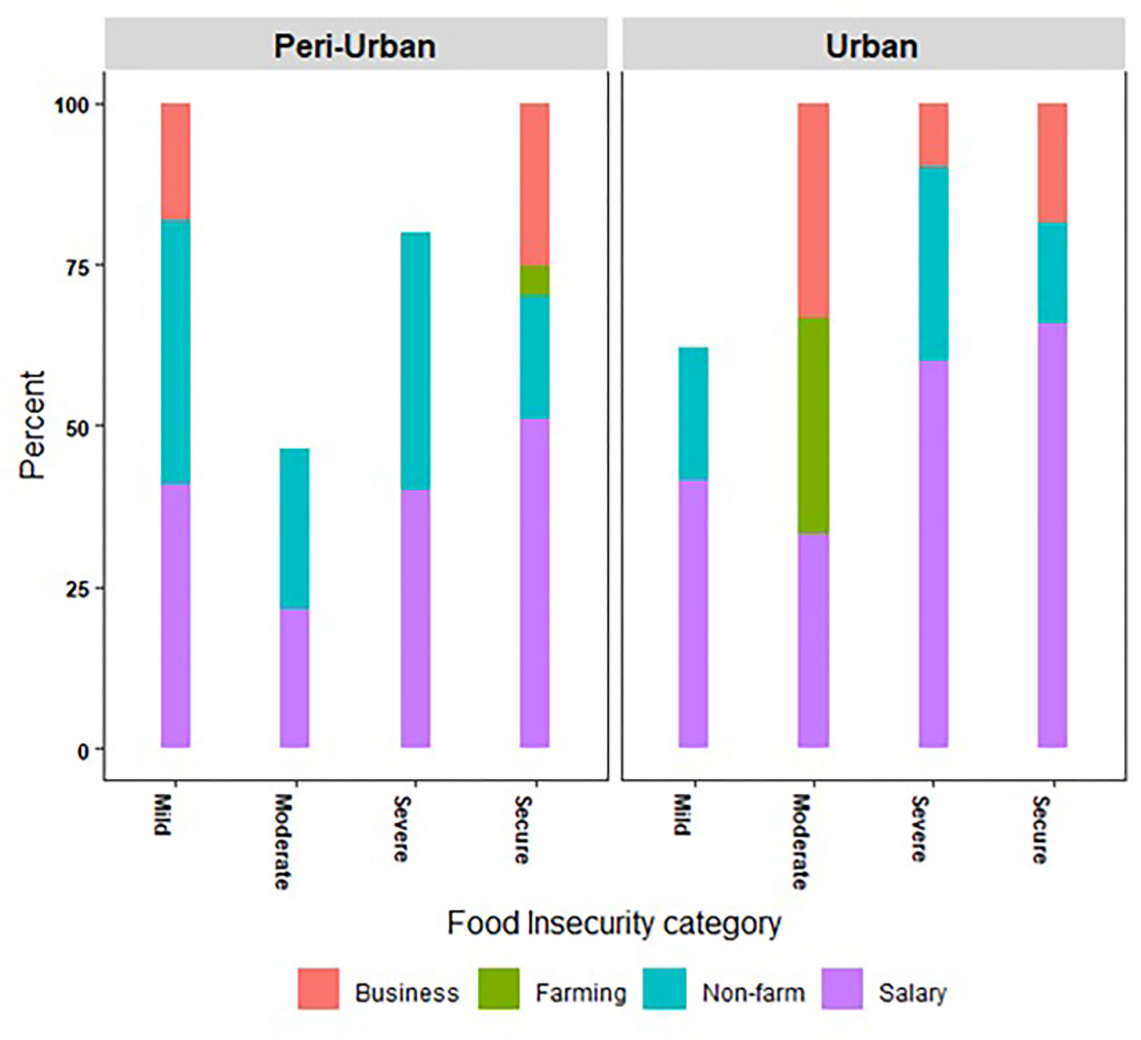
Household food insecurity status by type of employment in urban and peri-urban areas of Hyderabad.

The majority of households in our survey experienced a deterioration in their food security status during the pandemic. Households with moderate food insecurity status had the highest FIES score as well as number of unemployed days and lowest income. Around 25% of the households experienced mild food insecurity, and 17% experienced moderate food insecurity. Around 15% of the households reported an improvement in their food security status, while about 40% said it had worsened. Though the pandemic was a covariate shock, its idiosyncratic nature is evident from its differential impact on household food insecurity even in cases where the primary income earner belonged to the same class of worker. Overall, workers from peri-urban households bore a greater brunt of the impact than their counterparts in urban areas, as is evident from the higher incidence of worse food insecurity (both mild and moderate) among such households (Tables 2A,B, Figure 5).

**Table 2A:** Food insecurity status of sample households before and after COVID-19 outbreak in March 2020 in Hyderabad, India.

	**Pre-pandemic**						**Post-pandemic**					
	**Peri-urban**			**Urban**			**Peri-urban**			**Urban**		
	**Mild**	**Moderate**	**Secure**	**Mild**	**Moderate**	**Secure**	**Mild**	**Moderate**	**Secure**	**Mild**	**Moderate**	**Secure**
No.of households	22	19	151	12	13	108	49	33	109	34	21	76
FIES score	2.04	5.42	0.00	2.33	7.07	0.00	2.18	5.15	0.00	2.32	4.76	0.00
Proportion	6.76	5.84	46.46	3.69	4.00	33.23	15.07	10.15	33.53	10.46	6.46	23.38
**%Change**							**8.31**	**4.31**	**−12.93**	**6.77**	**2.46**	**−9.85**

*FIES score is the number of affirmative responses of the households to the Food Insecurity Experience Scale administered*.

**Table 2B:** Improvement/deterioration in household food security (in terms of FIES score) due to impact of COVID-19 outbreak in March 2020 in Hyderabad, India.

	**Improved (*n* =45)^†^**	**Deteriorated (*n* = 123)^**ℓ**^**
FIES score	0.82	3.47
Unemployed days	136	140
Family Size	5.00	4.00

† *Improved: Household whose food security status has improved in the pandemic period compared to the pre pandemic period*.

ℓ *Deteriorated: Household whose food security status has deteriorated in the pandemic period compared to the pre pandemic period*.

#### Determinants of Household Food Insecurity in the Post-pandemic Period

Based on a logistic regression model approach, Table 3 shows that income is negatively associated with all forms of food insecurity. We found that income and the type of employment of the primary income earner of a household are the determinants of a change in the food security status of a household. Since income stability is largely dependent on the nature of primary employment of a household (self-employment, regular salaried work, casual work, etc.), being employed in the private sector is associated with a lesser likelihood of household food insecurity. Similarly, residing in an urban area was associated with an increased likelihood of household food insecurity. Self-employed households living in urban areas seemed to face an increased risk of being food-insecure, a finding that could be attributed to the large income losses such households have suffered during and after the lockdown (Table 3).

**Table 3:** Determinants of household food insecurity in urban and pei-urban locations during the COVID-19 pandemic (March-November 2020).

	**Insecure**	**Mild**	**Moderate**	**FIES**
Intercept	5.137^**^	2.846	0.878	
	(2.047)	(2.076)	(2.529)	
Income	−0.554^***^	−0.433^**^	−0.315	−0.432^**^
	(0.207)	(0.211)	(0.259)	(0.171)
Unemployed days	0.004^*^	0.001	0.007^**^	0.005^***^
	(0.002)	(0.002)	(0.003)	(0.002)
**Place (Base category: Peri-Urban)**			
Urban	−1.406^*^	−1.726^*^	0.337	−0.870
	(0.77)	(0.896)	(1.013)	(0.688)
**Occupation (Base category: Casual Non-farm worker)**			
Farm	−1.101	0.120		−1.444
	(0.974)	(0.972)		(0.893)
Private sector	−0.975^**^	−0.299	−1.134^*^	−1.014^**^
	(0.479)	(0.501)	(0.667)	(0.43)
Public sector	−0.156	−0.267	0.562	0.213
	(0.684)	(0.719)	(0.875)	(0.609)
Self-employed	−0.877^*^	−0.0173	−1.219^*^	−0.828^*^
	(0.484)	(0.494)	(0.674)	(0.429)
Others	−0.022	−0.953	1.079	0.440
	(1.005)	(1.171)	(1.048)	(0.847)
**Place** **× Occupation**			
Urban × Farm				
Urban × Private sector	0.925	0.914	0.505	0.926
	(0.684)	(0.774)	(0.917)	(0.628)
Urban × Public sector	0.540	1.289	−0.789	−0.034
	(1.045)	(1.105)	(1.453)	(0.923)
Urban × Self-employed	1.581^**^	2.075^***^	−0.637	0.691
	(0.748)	(0.796)	(1.103)	(0.65)
Urban × Others	−0.069	1.936		−0.974
	(1.639)	(1.747)		(1.53)
**Coping strategies**			
**Place** **× Savings**				
Peri-Urban × Yes	−1.291^***^	−0.347	−1.982^***^	−1.324^***^
	(0.368)	(0.384)	(0.591)	(0.343)
Urban × Yes	−1.082^**^	0.214	−2.518^***^	−1.313^***^
	(0.488)	(0.547)	(0.796)	(0.477)
**Place** **× Loans**			
Peri-Urban × Yes	0.676^*^	0.221	1.186^*^	0.718^**^
	(0.386)	(0.389)	(0.624)	(0.353)
Urban × Yes	1.118^**^	0.548	1.122^*^	0.962^**^
	(0.435)	(0.462)	(0.59)	(0.388)
**Place** **× stored food**			
Peri-Urban × Yes	0.373	0.787^**^	−0.493	−0.079
	(0.374)	(0.377)	(0.524)	(0.332)
Urban × Yes	0.809^*^	1.157^**^	−0.336	0.323
	(0.483)	(0.551)	(0.669)	(0.453)
Observations	316	316	306	316
Pseudo *R*^2^	0.176	0.071	0.287	0.099
Akaike's Crit	393.099	370.362	237.258	871.183

*Standard errors are in parentheses*.

*** *p < 0.01*,

** *p < 0.05*,

* *p < 0.1*.

*Logit estimates reported. The dependent variable in column 2 is 1 if household is food-insecure and 0 if there is no food insecurity. The dependent variable in column 3 is 1 if household faces mild food insecurity and 0 if no food insecurity, or faces moderate or severe food insecurity. The dependent variable in column 4 is 1 if the household faces moderate and severe food insecurityand 0 if there is no food insecurity or mild food insecurity. Finally, the dependent variable in column 5 is FIES score 0–8, where ahigh number corresponds to high food insecurity*.

The results reflect that those employed in the private sector were less likely to experience food insecurity. Urban households were found to be at greater risk of food insecurity than peri-urban households with similar employment status. This possibly was due to the drastic reduction in job opportunities during the lockdown and the higher cost of living in the urban areas. Urban households, despite having better access to financial resources/services such as loans and savings, found it difficult to cope with the stress of food insecurity as can be seen from the positive association between urban dwelling and food insecurity.

The negative coefficients on savings could be attributed to the possibility that these households have lesser savings to tide over a food insecurity situation for long. Once the savings are spent, these households find themselves food-insecure (Table 3). The positive coefficients on loans show that access to finance was employed as a coping strategy by food-insecure households in both urban and peri-urban contexts. The positive association between self-employed urban households and food insecurity is plausibly due to the supply chain disruptions that impacted their business and the overall dampening of demand due to reduction in income.

#### Dynamics of Household Food Security

In assessing the food security status of households during the pandemic, we used data that we had collected before the onset of the pandemic (pre-pandemic food security status) as a benchmark against which to understand the changes that occurred during the 5-week-long lockdown and the three-phased relaxation of restrictions.

We found that the pre-pandemic food security status of a household based on the assessment using the first round of data collected in 2018 was a major determinant of its food security status in the lockdown-unlock period as well. This finding highlights the need to prioritize and target the already vulnerable households and ensure that they are covered by the assistance programs and social safety net schemes launched by various agencies in the aftermath of the outbreak. Savings are positively associated with improvement in household food security, while also being negatively associated with deterioration in food security status. This effect points to a likely correlation between private job holders who have greater income stability and also the inclination and opportunity to save more money and resources relative to, for example, non-farm workers. This proposition indeed finds resonance in the negative coefficients we found for urban households employed in the private sector. However, this benefit did not accrue to self-employed households, which endured deterioration of food security as they suffered large reductions in income besides having less access to savings (Table 4).

**Table 4:** Dynamics of household food insecurity ‘as assessedin terms of FIES scores.

	**Improved**	**Deteriorated**
Intercept	−0.388	2.712
	(2.702)	(1.985)
Income	0.715	−0.422^**^
	(0.622)	(0.213)
Unemployed days	−0.006	0.004^*^
	(0.009)	(0.002)
Precovid score	3.063^***^	−0.431^***^
	(0.694)	(0.108)
**Region (base category: Peri-Urban)**		
Urban	6.557	−0.989
	(5.687)	(0.789)
**Occupation**		
Farm	−0.799	−0.098
	(27.111)	(1.013)
Private sector	2.676	−1.15^**^
	(1.638)	(0.501)
Public sector	−0.072	−0.788
	(2.182)	(0.73)
Self-employed	1.516	−1.219^**^
	(1.955)	(0.509)
Others	4.739	0.635
	(5.263)	(1.247)
**Region** **× Occupation**		
Urban × Farm		
		
Urban × Private sector	−7.095	1.244^*^
	(4.468)	(0.71)
Urban × Public sector	−2.366	1.298
	(3.558)	(1.077)
Urban × Self-employed	−2.548	1.728^**^
	(4.28)	(0.781)
Urban × Others	−14.31	
	(165.654)	
**Coping strategies**		
**Region** **× Savings**		
Peri-Urban × Yes	6.212^***^	−1.349^***^
	(2.201)	(0.389)
Urban × Yes	4.338	−1.36^**^
	(4.763)	(0.553)
**Region** **× Loans**		
Peri-Urban × Yes	−0.787	0.781^*^
	(1.279)	(0.41)
Urban × Yes	−3.05	0.99^**^
	(1.99)	(0.451)
**Region** **× stored loans**		
Peri-Urban × Yes	0.480	0.380
	(1.076)	(0.396)
Urban × Yes	−3.141	0.267
	(4.791)	(0.522)
Observations	316	312
Pseudo *R*^2^	0.825	0.211
Akaike's crit	84.11	365.35

*Standard errors are in parentheses*.

*** *p < 0.01*,

** *p < 0.05*,

* *p < 0.1*.

*Logit estimates reported. Dependent variable in column 2 is 1 if household experienced improvement in food security status and 0 if there was no change in food security status, or faced deterioration in food security status. Dependent variable in column 3 is 1 if household experienced deterioration in food security status and 0 if there was no change in food security status, or experienced improvement in food security status*.

#### Coping Strategies

We found that households with different food insecurity status employed different coping strategies—depending on their access to such options. Households in both urban and peri-urban areas employed similar coping strategies to deal with the stress to their food security. Households in a state of mild food insecurity tended to fall back on their store of food as a coping mechanism. Households who were in a more intense state of food insecurity (see columns “moderate” and “FIES” in Table 3) tended to take loans or liquidate into savings to cope with the stress (Table 3). Households in the “mild food insecurity” category have relatively more access to stored food compared to other households. Similarly, those in the “moderate food insecurity” category have relatively more access to financial resources in the form of loans—from both formal and informal sources—but have the lowest access to savings. Households that experienced an improvement in their food security status have relatively high access to financial resources both in the form of loans and savings besides being protected by various social safety schemes. On the other hand, households that experienced a deterioration in their food security status have better access to loans compared to savings (Table 5).

**Table 5:** Household access to coping strategies based on FIES scores.

	**Percent households with access**				**Percent households without access**			
**Coping strategy**	**Mild**	**Moderate**	**Improved**	**Deteriorated**	**Mild**	**Moderate**	**Improved**	**Deteriorated**
Loans	66.27	80.36	64.44	71.54	33.73	19.64	35.56	28.46
Savings	43.27	16.07	71.11	30.08	56.63	83.93	28.89	69.92
Stored food	57.83	41.07	28.99	52.03	42.17	58.93	71.11	47.97
Gov Aid	86.75	87.5	93.33	87.8	13.25	12.50	6.67	12.20

*Mild and Moderate denote actual food insecurity status of the household in the pandemic period.Improved and deteriorsated denote the dynamics in the food security status of the household in the pandemic period compared to the pre pandemic period*.

Households whose food security status deteriorated (Table 2B) had the highest number of unemployed days on average. Households experiencing “moderate food insecurity” have less access to savings like those in the “deteriorated” category. More than 70% of the households in the “moderate” and “deteriorated” categories borrowed money to cope with the exogenous shock of the pandemic. Households in the “mild food insecurity” category employed stored food as a coping strategy to a greater extent as they have that tendency to store food more than other kind of households. Nearly 60% of the households experiencing “mild food insecurity” lacked access to savings.

## Discussion

### Economic Impacts and Impacts on Food Security

There has been a severe contraction of labor demand in India that has materialized unevenly across different occupations and skill levels. According to the Center for Monitoring Indian Economy,^9^ the labor force participation rate fell to an all-time low in March 2020, and the unemployment rate rose sharply. Employment slumped by 9 million jobs, going from 443 million in January 2020 to 434 million in March 2020. This decline was the result of a fall of 15 million (from 411 million to 396 million) in the number of employed people and a 6 million rise (from 32 million to 38 million) in the number of unemployed people in March 2020.

As the COVID-19 lockdown progressed, 80% of India's informal workers lost their jobs (42). The livelihoods of daily workers, street vendors, small enterprises, and retail traders came to a complete stop for various reasons (43). More than 50% of informal workers and their families are estimated to have been pushed into poverty due to the reduction in labor incomes triggered by the lockdown (44). In the urban regions of the state of Telangana—whose capital is Hyderabad—the labor force participation rate and the greater unemployment rate were 40.47 and 5.20%, respectively, between September and December 2020 (45).

Being the major city in Telangana, Hyderabad was an interesting case to examine the trends in labor force participation in and around the city. The trends observed in our study can be attributed to the restrictions imposed on physical mobility in the city when the lockdown was announced on March 24, 2020. For people who live in the peri-urban areas around the city and commute to work in the non-farm sector everyday, this cut off access to employment. The mass layoffs and the closure of many small businesses in and around the city further added to these difficulties. The results of our study indicate that the lockdown's impact on livelihoods was more severe in peri-urban areas than in urban areas. This is consistent with the general consensus in India that the pandemic hit small businesses, daily-wage earners, and low-wage earners, leaving them with no jobs or reduced incomes (46). These findings are also in line with the macro trend observed at the national level: the national unemployment rate increased to 26% in April 2020, before easing to 19% in the subsequent months (45).

It is reasonable to expect that income losses across sectors in the urban areas resulted from the closure of businesses across sectors, especially small businesses. Such a major impact on livelihoods leads to reduced economic access to resources and services and thereby increases the risk of food insecurity. In addition, food accessibility in urban areas is largely the result of food affordability (47). The increase in staple cereal prices due to the COVID-19 outbreak in Asia has started to impact prices in local markets (48). Reduced purchasing power due to a drop in household incomes impacts their food security.

Besides reduction in income, urban consumers were also affected by the disruption of long-distance food supply chains, which resulted in product non-availability and higher retail/online prices. There was an 8% decline in the availability of fruits and vegetables and a 14% decline in the availability of edible oils in three of India's metropolitan cities immediately after the lockdown was imposed. The prices of major food items—constituting more than 25% of the urban food consumption basket—spiked immediately after the lockdown was announced in March 2020: pulses by 6%, edible oils by 3.5%, potatoes by 15% and tomatoes by 28% (49, 50). Economic access to food was constrained due to reduced income, restrictions on physical movement and restricted consumer access to affordable food markets, as reflected in the Composite Consumption Behavior Change Index (CCBCI) (51).

However, not all non-farm workers were equally affected by the lockdown. The differential impact observed across the informal sector could be attributed to the two separate branches of employment that exist within the informal sector: One group comprises those who operate/work informally within informal enterprises and those outside of informal enterprises Valodia et al. (52). The second group includes casual laborers, domestic servants, and other forms of labor-intensive employment, who tend to rely on cash income. Those in the latter group may be the most at risk of food insecurity as they face the challenge of inconsistent income. Households with members employed in the formal sector, where income is regular, do not have the same risk of food insecurity (53).

The findings of our study show that the type of employment in which the primary income earner of a household is engaged and the income it earns for the household are the determinants of any change in the food security status of the household. The sensitivity of food consumption to income changes, particularly labor income, is well-established in literature (54, 55). Liquid savings and access to credit influence heterogeneous consumption responses to income shocks (56). Our results support these findings as households that are primarily employed in sectors that provide better job security and greater stability in income relative to non-farm work which is characterized by high levels of insecurity both in terms of job and income. As a result, they are less likely to face more intense levels of food insecurity (moderate category). Overall, our results support the findings of Egger et al. (57), who report that both negative income shock and income level affect the predicted probability of households facing food insufficiency or insecurity.

Regular salaried employment such as in the private sector helps households improve their food security status gradually compared to households primarily employed in the non-farm sector as casual labor. Most of the households in our sample went from being food-secure in the pre-pandemic period to being food-insecure since the outbreak in March 2020. Relatively, private sector jobs offer higher job security and other optional benefits than jobs in the non-farm sector. Private sector jobs also offer better income stability compared to non-farm jobs. With relatively higher income, households are better equipped to make savings, which can be utilized to withstand an unexpected shock corroborating with findings of Gjerston, 2016 (58). Our findings support similar findings by Kesar et al. (59), who reported that self-employed households were better off in terms of food security, especially in urban areas despite facing job losses. Furthermore, our results also corroborate the findings of literature, our results show that households that are able to overcome short-term liquidity constraints by borrowing seem to smoothen food consumption and are less likely to be food-insecure, implying that they can mitigate the risk of becoming food-insecure (58, 60–63).

Households that were already food-insecure before the pandemic experienced higher levels of food insecurity on a relative scale. The findings of Gaintan-Rossi et al. (64) support our finding that economic shocks more strongly affect households that were already vulnerable prior to the shock. Though households did use savings to cope with the exogenous shock of the pandemic, they probably did not have sufficiently large savings to sustain their food security for an extended period. Once the savings dried up, they were not in a position to access adequate food.

Our findings also show that there exists a relationship between low food security and households whose members are employed as casual workers (65). This link could be attributed to the implicit challenges present in the informal sector stemming from the absence of formal regulations. The multiple challenges faced by those employed in the informal sector are well-documented in the literature (66–69). The difficulties faced by a household in getting an assured employment that provides a sustained livelihood is one of the primary challenges in achieving food security in the urban areas. Food accessibility in urban areas is largely a result of food affordability (48).

### Coping Strategies

The coping strategies observed by our study corroborate with patterns observed in other developing countries and Low and Middle Income Countries (LMICs). Households have used formal and informal borrowing as a strategy to meet immediate expenses (70–73). Lack of access to such a coping strategy is reported to increase the likelihood of a household being food-insecure (74, 75). Also, the fact that most of the households' food security has deteriorated implies that they also relied on and employed both food-based coping strategies of reducing the quantity and quality of food consumption and financial coping strategies to tackle the stress to their food security caused due to an income shock (76). Since a large portion of the total household income goes toward food purchase and consumption in urban areas, these findings are expected. The monthly percapita food consumption expenditure in the urban regions of the study location (Hyderabad district) as per the 68th national sample survey stood at INR 1196.78 which was higher than the state avaerage in 2011–12. The inflationary pressure as reflected by the CPI and food inflation during the lockdown and the subsequent months of phased withdrawl were high at 6.6 and 9.1%, respectively, in 2020–21 in the country which had a huge impact on affordability. The impacts of high food inflation on consumption during the period were reflected in the CCBCI as elucidated in the above section on economic impacts. Our results on increased prevalence of food insecurity in the post pandemic period resonates and corroborates with findings of Srivastava and Sivaramane (77) who estimate reduction in the overall food expenditure between 4.98 and 21.34% compared to the pre pandemic period.

## Conclusions

The COVID-19 pandemic has negatively impacted food systems and food security in the Hyderabad region of India. The impact of the pandemic on the households has been heterogenous.Households have experienced both idiosyncratic and covariate shocks (78, 79). The findings of our study reflect the transitory nature of food security in the region as a result of the shock. Household food security dynamics are largely influenced by the sector in which the main earning member of a household is employed, income, and access to different coping mechanisms. The use of coping strategies seems to depend on their availability and accessibility to the household. Our results reaffirm the significance of employment oppurtunities and savings for the poor to circumvent unexpected shocks.Our study thus underscores the need for policy that promotes saving behavior among the poor in addition to ensuring them secure employment oppurtunities that provides stable income. These results confirm the importance of savings for poor households and underline the crucial role policies can play to support savings and ensure stable incomes through secure employment opportunities. Ex-ante or forward-looking risk and vulnerability analyses are essential for targeting and implementing risk mitigation interventions. Since our study is based on cross-sectional data, the findings of the study are not generalizable to a wider context. We recommend such assessments in the future which will help improve the preparedness of society and communities to cope with such unprecedented situations through targeted efforts.

### Limitations

Using remote/telephonic survey as a methodology for data collection during the pandemic period is not unproblematic. Even though these surveys are cost effective compared to face to face interviews, the most important issue is the reduction in the response rate, i.e., the loss in the sample due to either telephone not working, change in the contact numbers given earlier, and repondents refusal to take the survey. Other issues with phone-based that were observed include infrastructural constraints in some settings (e.g., electricity and mobile connectivity issues), length of the interview and concerns around ensuring participant privacy. Thus, even though phone-based surveys are considered suitable in many settings, these limitations appear and have to be addressed.

Another limitation of the study is its inability to apply a gender lens to this understanding of food security during the pandemic. There were cases when women could not be contacted during our telephonic survey as many of them did not have access to a personal mobile phone, or their husbands were not interested in their wives participating in the telephonic survey. Further, women we contacted also said their care responsibilities had increased due to the pandemic and hence they were not available to participate in the survey. A gendered understanding would have brought out interesting insights on the differential impacts of the pandemic.

## Data Availability Statement

The raw data supporting the conclusions of this article will be made available by the authors, without undue reservation upon request.

## Ethics Statement

The studies involving human participants were reviewed and approved by ICRISAT. The patients/participants provided their written informed consent to participate in this study.

## Author Contributions

RP: conceptualization, methodology, data curation, investigation, formal analysis, writing—original draft, and writing—review and editing. SN: conceptualization, methodology, writing—original draft, and writing—review and editing. PJ and KK: data curation, investigation, formal analysis, and writing—original draft. C-JL and AA: conceptualization, methodology, writing—review and editing, and funding acquisation. All authors contributed to the article and approved the submitted version.

## Funding

This study was supported by funding (Grant Number: 2016-00350) from the Swedish Research Council for Environment, Agricultural Sciences and Spatial Planning (FORMAS).

## Conflict of Interest

The authors declare that the research was conducted in the absence of any commercial or financial relationships that could be construed as a potential conflict of interest.

## Publisher's Note

All claims expressed in this article are solely those of the authors and do not necessarily represent those of their affiliated organizations, or those of the publisher, the editors and the reviewers. Any product that may be evaluated in this article, or claim that may be made by its manufacturer, is not guaranteed or endorsed by the publisher.

## Footnotes

^1^Food Insecurity Experience Scale | Voices of the Hungry | Food and Agriculture Organization of the United Nations (fao.org).

^2^A sub-administrative division commonly used in India and some Asian countries.

^3^Anganwadis are rural child care centers in India. They were started by the Indian government in 1975 as part of the Integrated Child Development Services (ICDS) program to combat child hunger and malnutrition. Each center is managed by an anganwadi teacher.

^4^The elected head of a village assembly or gram sabha.

^5^ASHA = Accredited Social Health Activist.

^6^Census and Survey Processing System: https://www.csprousers.org/.

^7^ https://www.kobotoolbox.org/

^8^Refers to the nature of primary employment of the main income earner of the household: self-employment, salaried work which includes private and public sector, casual work, etc.

^9^ https://unemploymentinindia.cmie.com/
