# Supplementary material for: COVID-19 Impact on Household Food Security in Urban and Peri-Urban Areas of Hyderabad, India
Source: Front Public Health. 2022 May 13;10:814112. doi: 10.3389/fpubh.2022.814112 (PMC9136225; doi:10.3389/fpubh.2022.814112)
Supplement: Supplementary file 1 [file Data_Sheet_1.pdf]

APPENDIX 1

Name of the selected locations				
**Grid No**	**Quadrant 1**	**Quadrant 2**	**Quadrant 3**	**Quadrant 4**
23	Ramachandra puram	Sri Sainagar (Bachpally)	Lingampally	KPHB Phase 7
19	Mallapur	Pocharam	Sai nagar (Nagole)	Gowrelly
17	Madhapur	Begumpet	Tolichowki	Afzalgunj
10	Tolichowki	Afzalgunj	Langerguda/Gaganpahad	Chandrayangutta

APPENDIX 2

2A. HOUSEHOLD SURVEY QUESTIONNAIRE—ROUND 1

Informed consent: *It is necessary to introduce the household to the survey and obtain the consent of all prospective respondents to participate, in this case the head of the household, the mother, child (3-5 years) and adolescent male or female (11–18 years). Be sure to obtain their verbal consent before interviewing them.*

Thank you for the opportunity to speak with you. We are a research team from ICRISAT. We are conducting a survey to learn about your livelihood, food security, food consumption, nutrition and well-being of households in this area. Your household has been selected to participate in an interview that will include questions on topics such as food consumption and nutrition. The survey includes general questions about the household, and questions about individuals within your household, if applicable. These questions will take approximately 45 minutes to 1 hour to complete and your participation is entirely voluntary. If you agree to participate, you can choose to stop at any time or to skip any questions you do not wish to answer. Your answers will be completely confidential; we will not share information that identifies you with anyone. After entering the questionnaire into a database, we will destroy all information, such as your name, thatcan link these responses to you.

Please ask the participants if they consent to participating in the study and put their signature or thumb impression below:

Household Unique ID No:	Date of interview:
Name (adult male):	
Name (adult female, and take consent on behalf of the child):	
Name (adolescent):	

The enumerator responsible for the interview certifies having read the statement out to the participant and that they have consented to the interview. The enumerator also pledges to conduct this interview as indicated in the instructions and inform the supervisor of any problems encountered during the process.

<u>Informed consent (written)</u>

<u>క్రిందనున్న విషయాలను ప్రత్యుత్తరమిచ్చే వారికి చదివి వినిపించి, సర్వే కొరకు వారి అనుమతి పొందండి.</u>

ఇది ఎన్నిక కాబడిన గృహములోని వారికి సర్వే గురించి పరిచయం చేయటానికి మరియు పాల్గొనే ప్రతి ప్రత్యుత్తరమిచ్చే వారి యొక్క సమ్మతిని పొందటానికి అవసరం. ప్రత్యుత్తరమిచ్చే (ఉదా: స్త్రీ పునరుత్పత్తి వయస్సు)ఆమెను ఇంటర్వ్యూ చేయడానికి ముందు సమ్మతిని పొందాలి.

మీతో మాట్లాడే అవకాశం కల్పించినందుకు ధన్యవాదములు. మేము ఇక్రిశాట్ నుండి వచ్చిన పరిశోధన బృంద సభ్యులం. వ్యవసాయం, ఆహార భద్రత, ఆహార వినియోగం, పోషకాహారం మరియు ఈ ప్రాంతంలో గృహాల శ్రేయస్సు గురించి తెలుసుకోవడానికి మేము ఒక సర్వే నిర్వహిస్తున్నాము. మీ ఇంటిని ఇంటర్వ్యూలో పాల్గొనడానికి ఎంపిక చేయబడింది, ఇందులో మహిళలు మరియు పిల్లల ఆహార వినియోగం మరియు పోషణ వంటి అంశాలపై ప్రశ్నలు ఉంటాయి. ఈ సర్వేలో సాధారణంగా గృహాల గురించి మరియు మీ ఇంటిలోని వ్యక్తుల గురించి ప్రశ్నలు, వర్తిస్తాయి. ఈ ప్రశ్నలు మొత్తం సుమారు 45 నిమిషాలు నుండి 1 గంటకు వరకు పడుతుంది, మరియు మీ భాగస్వామ్యం పూర్తిగా స్వచ్ఛందంగా ఉంటుంది. మీరు పాల్గొనడానికి అంగీకరిస్తే, మీరు ఎప్పుడైనా ఆపడానికి ఎంచుకోవచ్చు లేదా మీరు సమాధానం ఇవ్వకూడదనుకున్న ప్రశ్నలను దాటవేయవచ్చు. మీ సమాధానాలు పూర్తిగా రహస్యంగాఉంటాయి. మిమ్మల్ని ఎవరైనా గుర్తించే సమాచారాన్ని మేము భాగస్వామ్యం చేయము. డేటాబేస్ లోకి ప్రశ్నాపత్రాన్ని ప్రవేశించిన తర్వాత, మీ ప్రతిస్పందనలు లింక్ చేసే మీ పేరు వంటి వాటిని మేము తొలగిస్తాము.

A. HOUSEHOLD IDENTIFICATION

1. **Region:** Peri-urban/Urban (circle what is applicable)

**Name of the ward/locality**		**Name of the district**<br>Hyderabad/Rangareddy
**Complete postal address**		**Landmark to identify the house**
**GPS coordinates (please enable in the tab)**		

**A01**. Start time of interview (hh:mm)	☐☐ : ☐☐
**A02**. Household identification	☐☐☐☐☐☐☐☐☐☐
**A03**. Name of head of the household<br><br>**Name**: ....<br><br>**Surname:**...	
**A04.** Type of household:<br>1 = Male and female adult ; 2 = Female adult only; 3=Male adult only	
**A05**. Main religion of the household (enter code)<br><br>1=Muslim; 2=Hindu; 3=Christian; 4=Buddhist; 5=Navbudhha; 6=Others	

**A06**. Main language of the household<br>Telugu = 1; Marathi = 2; Hindi = 3; Other (specify) = 4	
**A07**. Main caste of the household	
**A08**. Caste category:<br>FC = Forward Caste; BC = Backward Caste; SC = Scheduled Caste; ST = Scheduled Tribe; NT = Nomadic Tribe	
**A9.** From which market(s) do you source your goods? (list all)	
**A10**. Does your locality have a weekly market for vegetables?<br>Yes/No	
A11. If yes, what is the market day of your locality – Monday, Tuesday, etc	
A12. Do you buy vegetables from the weekly market?<br>Yes/No	
A13. Name of the enumerator<br>…………………………………………………………………..	Employee #
A14. Date of first visit (dd/mm/yyyy):	☐☐ / ☐☐ / ☐
A15. Date of subsequent visit (dd/mm/yyyy):	☐☐ / ☐☐ / ☐
**A15.** Reason for subsequent visit:<br>..	

**A16a.** Mobile phone numbers: Head of the household	
**A16b.** Aadhaar card number of head of household	
**A17a.** Mobile phone numbers: Spouse	
**A17b.** AADHAAR card number of spouse:	

**A18.** End time of interview (hh:mm):	☐☐ : ☐☐
**A19.** General remarks/additional notes	

HOUSEHOLD ROSTER

ID CODE	Name of household member<br><br>[start with head of household, continue with the other members of the household]	**A20a: Sex**: M = Male; F = Female<br>**A20b: Status:**<br>Married = 1; Single/ Never married = 2; Separated = 3; Divorced = 4; Widowed = 5<br>**A20c:** If married, age at marriage (completed years)			Relationship to the head of the household	Age<br>(in completed years)*	Can you read and write?	Is [NAME] currently attending school?<br>1 = Yes; 2 = No<br>If Yes, goto A26;<br>if No, goto A25 and continue	Has [NAME] ever attended school?<br>Y= Yes<br>N= No<br>If Yes, goto A26;<br>if No, goto A27	What is the highest level of education completed by [NAME]?	Whether member of any organization<br>Y/N;<br>Name of the organization	Did any member leave the village/ native place?<br>1 = Yes; 2 = No
	**A19**	**A20a**	**A20b**	**A20c**	**A21**	**A22**	**A23**	**A24**	**A25**	**A26**	**A27**	**A28**
1					Hea							
2					Spo							
3												
4												

5												
6												
7												

**A21: Relationship to head of household**<br>1 = Head of household<br>2= Spouse/partner<br>3 = Son/daughter<br>4 = Son-in-law/daughter-in-law<br>5 = Grandson/granddaughter<br>6 = Mother/Father<br>7 = Brother/sister<br>8 = Nephew/niece	9 = Nephew/niece of spouse<br>10 = Cousin of primary respondent<br>11 = Brother/sister-in-law<br>12 = Mother/father-in-law<br>13 = Cousin of primary respondent's spouse<br>14 = Other relative<br>15 = Servant/maid<br>16 =Laborer<br>17 = Other relationship (Specify)	**A23: Literacy**<br>1 = Cannot read and write<br>2 = Can only sign (write)<br>3 = Can only read<br>4 = Can read and write	**A26: Education level**<br>1 = No school<br>2 = Primary level classes 1-5<br>3 = Secondary level classes 6-10<br>4 = Technical or vocational training<br>5 = Intermediate<br>6 = Graduation /University (BSc/BA/B.Com /B.Tech)<br>7 = Post [illegible]	**A27: Organizations**<br>1 = SHG<br>2= PACCS<br>3 = Farmer cooperative<br>4 = Gram Panchayat<br>5 = MPTC<br>6 = Education committee<br>7 = Water Users Association	

YEARLY INCOME OF THE HOUSEHOLD

TYPE OF HOUSEHOLD/CARD	APL/BPL	Others

	(APL = Above poverty line; BPL = Below poverty line)	
**Source of income**	**Annual Income (₹)**	**Remark**
1- Farming		
2- Livestock		
3- Wages		
3.1 On farm		
3.2 Non-farm		
4. Fish farming		
5. Salary		
6. Business		
7. Other (specify)		

**FOOD INSECURITY EXPERIENCE SCALE**			
Now I would like to ask you some questions about your food consumption. During the last 12 months, was there ever a time when:			
**Household food insecurity**	**Response**	**Response codes**	
1. You were worried you would run out of food because of a lack of money or other resources?		0 No<br>1 Yes	98 Don’t Know<br>99 Refused
2. You were unable to eat healthy and nutritious food because of a lack of money or other resources?		0 No<br>1 Yes	98 Don’t Know<br>99 Refused
3. You ate only a few kinds of foods because of a lack of money or other resources?		0 No<br>1 Yes	98 Don’t Know<br>99 Refused

4. You had to skip a meal because there was not enough money or other resources to get food?		0 No<br>1 Yes	98 Don’t Know<br>99 Refused
5. You ate less than you thought you should because of a lack of money or other resources?		0 No<br>1 Yes	98 Don’t Know<br>99 Refused
6. Your household actually ran out of food because of a lack of money or other resources?		0 No<br>1 Yes	98 Don’t Know<br>99 Refused
7. You were hungry but did not eat because there was not enough money or other resources for food?		0 No<br>1 Yes	98 Don’t Know<br>99 Refused
8. You went without eating for a whole day because of a lack of money or other resources?		0 No<br>1 Yes	98 Don’t Know<br>99 Refused

APPENDIX 3: IMPACT OF COVID-19 ON HOUSEHOLD FOOD SECURITY IN URBAN AND PERI-URBAN LOCATIONS OF HYDERABAD, TELANGANA, INDIA

Online Consent

I am an adult and I consent to taking this survey.

The survey will help in understanding the impact of the COVID-19 pandemic and the information we collect can be used to inform government policies and relief measures. I would like to ask you some questions on this topic. There is no known risk to this survey. If you do not wish to answer any question or wish to terminate the interview at any point, please do let us know. We will provide you information on the virus and the relief measures initiated by the government. I anticipate that this survey/interview will take 15 minutes to complete. All information that you share with us will remain confidential and any data and research findings shared in the public domain will be anonymized. The information will not be used for commercial purposes Your participation is strictly voluntary and you may refuse to participate at any time. If you have questions later, please contact me at XXXXX . If you would like to speak to our survey supervisor, you can call them at XXXXXXX. Do you understand the purpose and nature of this study and agree to voluntarily participate in it by answering the questions in the survey?

If “**Yes**”: I appreciate your willingness to help with this project.

If, “**No**”: “Is this a bad time? Could you please let me know what would be a good time to talk?”

OR Still I do not consent taking this survey (skip to end)

100. Demographic Roster

101. Country: India

102. State: Telangana

103. Area: Urban area/Peri-urban area

104. Location:

- Mallapur
- Pocharam
- Nagole
- Gowrelly
- Gaganpahad
- Chandrayangutta
- Ramachandrapuram
- Bachpally
- Lingampally
- Kukatpally
- Madhapur
- Begumpet
- Afzalgunj
- Tolichowki

105. Household Identification ID:

106. Date of interview:

107. Time of interview:

108. Mobile phone number:

109. Name of the respondent:

110. Gender of the respondent: Male/Female

111. Occupational status of the adult male in the household:

112. Occupational status of the adult female in the household:

113. What is the relationship with the main income earner of the household?

a. Self

b. Spouse

c. Parent/Parent-in-law

d. Brother/sister/Brother-in-law/sister-in-law

e. Son/daughter/Son-in-law/daughter-in-law

f. Other family member

g. Others

114. Do you or someone in your household own land that you cultivate or can cultivate?

a. Yes

b. No

114(a). If yes, how much land (in acres) do you own?

Specify the numerical value

115. How many members are living in your household at present?

Specify the numerical value

200. Impact on livelihoods

200. A Baseline Information (February) Now I am going to ask you some questions about what you were doing in the month of February, i.e., the month before the lockdown. Please try and recall:

200a. Approximately, what was the total income of all members of the household for the month of February? [Include any remittances (in INR) that were received]

a. Less than 2,000

b. 2,000 to 5,000

c. 5,000 to 10,000

d. 10,000 to 20,000

e. More than 20,000

200b. In the month of February, how many members in your household were earning an income?

201. Describe the kind of work you do.

202. What was your primary activity status for the month of February?

a. Self-employed without any employees

b. Self-employed with one or more employees

c. Regular salary worker

d. Casual worker

e. NREGA worker

f. Unpaid helper in family business/farm

g. Unemployed

h. Student

i. Homemaker

j. Retired/Disabled

203. In which sector did you primarily work in the month of February, (that is, the sector from which you derived majority of your income):

a. Agriculture/forestry/fishing/animal husbandry

b. Mining

c. Manufacturing, without construction

d. Construction

e. Retail and wholesale trade

f. Hotels /Restaurants

g. Transport

h. Public Service Administration/Government service

i. Education

j. Health

k. Other service providers

204. What was the average DAILY wage rate at which you were working in the month of February?

205.A. How many days did you work in the month of February? [Enter 0 if they did not work any day]

205.B How many weeks did you work in February?

a. Less than one week

b. One week

c. Two Weeks

d. Three Weeks

e. All of the month

206. How much did you earn in your business in the month of February?

207. On an average, in any given week in February, how many people did you employ (for wages) in your farm/business?

208. What is the MONTHLY salary that you received in the month of February? [Enter 999 if don't know, 888 if refused to respond]

209. Are you usually paid via a contractor or directly by your employer?

a. Directly by employer

b. Via a contractor

c. Don't know

210 B: Labor Force Participation since lockdown (March 24, 2020)

210. Now, I am going to ask you some questions about what you have been doing since March 24 when the lockdown was announced:

Period	211. What were you doing	212. If < (211<6, activity status: If not unemployed or Not out	213. <211<6 Activity Status: If not unemployed or NOT out	214.<If (211=4Casual Worker)> On average, what was your **daily wage**	215_1. If <211=1/2/5 &212=1> [Enter 0 if they did	215_2. <If 211=1/2 & 212=1 & 215_1=1>

	in this period	of labor force)<br><br>What sector were you employed in? (refer sector codes)	of labor force><br><br>How many **days** did you work during this period from March 24 to today?	**rate** during this period?	not earn anything]<br><br>What were total earnings after March 24 till today?	On average, what was your weekly earnings during this period?
Lockdown period: Phase1:4 (24 March-31 May2020)						
Unlock period: Phase 1:2 (1 June-31 July 2020)						
Unlock period: Phase 3:4 (1 August-30 September 2020)						
Unlock period: Phase 5:6 (1 October-30 November 2020)						
Source: Lockdown and unlock periods in India. https://en.wikipedia.org/wiki/COVID-19_pandemic_lockdown_in_India						

216. If there was no lockdown, what were you expecting to do during this period?

217. Do you have crop/agricultural produce/poultry/fish with you right now that you are not able to sell due to the lockdown?

a. I am not able to harvest produce

b. I am not able to sell my produce

c. I was able to sell but at reduced price

d. I was able to sell at regular or higher price

e. I did not have any produce to sell

218. Why have you not been able to sell this?

a. No workers/machines available

b. Not able to go to market - no transport facilities

c. No buyers

d. Other, specify

219. How much money were you expecting to receive from the sale of this produce (in INR)? Enter 999 if don't know, 888 if refused to respond.

220a. How much less did you earn than what you expected?

a. Less than half

b. About half

c. More than half

220b. How much loan/credit you may need to do agriculture in coming kharif (rainy) season?

a. None

b. Less than Rs 5000

c. Rs 5000 to Rs 10,000

d. Rs 10,000 to Rs 20000

e. More than Rs 20,000

221. Did you have to lay off any of your workers since the lockdown?

a. Yes

b. No

c. Do not want to reveal

222. Were you/will you be able to pay wages to your employees since the lockdown?

a. Yes, I paid/will pay full wages

b. Yes, but only partial wages

c. No, I was not/will not be able to pay any wages

d. Do not want to reveal

223. Since the lockdown, has there been a decrease in the salary you are paid or that you expect to receive?

a. No, my salary has not changed;

b. Yes, I have been asked to take a reduction in salary payment this month;

c. Yes, I will not be paid for this month;

d. Don’t know

e. No, but my salary hike has been withheld

224. What was your monthly salary during the lockdown period (in INR) [Enter 999 if don’t know, 888 if refused to respond]?

225. Since the lockdown, have you received wages for the work done since?

a. Yes, received in full

b. Yes, received in part

c. No, did not receive

d. Do not want to reveal

226. Is there any payment (for sales/wages/salary) due to you prior to the lockdown that you have not received?

a. Yes

b. No

c. Don’t know

227 C. Access to Savings and Credit

227. How long will you have money to be able to pay for essentials if the current situation continues?

a. 2 days or less

b. Less than a week

c. Up to a week

d. 10 days

e. 2 weeks

f. 1 month

g. More than 1 month

228. Have you taken any loans to cover daily personal expenses due to the coronavirus?

a. Yes

b. No

229. Who did you take the loan from?

a. Bank

b. Cooperative Society

c. Self-Help group

d. Moneylender

e. Friend/Family

f. Contractor/Employer

g. Other

230. What amount of loan did you take? (Enter 999 if person doesn’t know, 888 for refused to respond)

231. Do you have any other loans taken before the pandemic that are outstanding?

a. Yes

b. No

232. < If 230 == a> Will you be able to repay instalments on that loan?

a. Yes

b. No, I got an extension on loan repayment

c. No, I have to pay but don’t have the money

300 Health Impact

301. Since March 1, has anyone been tested for Covid-19 in your household?]

- Yes, once
- Yes, twice
- Yes, more than two times
- No, my doctor recommended it but there weren't any tests available
- No, I have not had any symptoms requiring a test]

301a. If answer = yes to previous question: was the outcome positive on at least one occasion?

- Record the numerical value of the coronavirus test identification number
- Record the name of the hospital
- I prefer not to answer

301b. If ‘test = positive’: Which of the following symptoms have you experienced? (check all that apply)

- Fever
- Dry cough
- Productive cough
- Difficulty breathing
- Sore throat
- Headache
- Muscle pain
- Loss of sense of smell
- None of these symptoms
- I prefer not to answer

302. How long have you had these symptoms?

- I don’t have these symptoms
- 1 - 3 days
- 4 - 7 days

- 8 - 13 days
- 14 or more days
- I prefer not to answer

303. If test = positive: were you able to isolate yourself from other members in your home?

    - Yes
    - No

304. Are you a member of any of these risk groups? Check all that apply

- Hypertension
- Diabetes
- Cardiovascular diseases
- Respiratory illnesses
- Cancer
- Smoker
- Pregnant
- Health worker
- Not in a risk group
- I prefer not to answer

400. Impact of social safety net schemes/programs

401. Are you able to purchase your basic food essentials at this time?

a. Yes

b. Yes, but lesser quantities than usual (before the pandemic)

c. No

402. < If 401 = b/c > Why not?

Check all that appllies

a. Not available in the shop

b. I don’t have the money to buy these things

d. I am not able to go out to purchase these things

403. Is your household consuming the same amount of food as before the lockdown?

a. Yes, same as before

b. Eating less grains and vegetables as compared to before

c. Eating only one meal on many days

404. Do you live in a rented house/hostel/flat/temporary accommodation?

a. Yes

b. No

405. Do you have/expect to have enough money to pay the next rent?

a. Yes, I will be able to pay rent

b. No, I won’t be able to pay rent

406. Do you have a ration card that you can use where you live?

a. Yes, BPL/AAPY/priority card

b. Yes, APL or other card

c. Yes, don’t know the type of card

d. No

e. Don’t know

407. Have you received free rations or increased rations after the lockdown?

a. Yes from a government ration shop

b. Yes from other sources 12

c. Tried to procure from ration shop but was unable to do so since the announcement

d. Have not tried /did not need to access it

408. How much grain has your household received from the government in the lockdown period as relief (in kgs)?

409. Do you or anyone in your family have a bank account?

a. Yes

b. No

410.1. Is your household eligible for PM Kisan Yojna?

a. Yes

b. No, my household is not eligible

c. No, I don't know whether I am eligible

d. No, I don't know about this scheme

411. How much did you or your household receive as transfer into your account as part of PM Kisan Yojna Scheme?

(777 not eligible, 999 do not know, 888 refused to respond)

411.1. Do any women in the household have a Jan Dhan account?

a. Yes

b. No

c. Don't know

411.2: How much did you or a household member receive as transfer into your Jan Dhan account? (999 do not know, 888 refused to respond)

PLEASE NOTE: Recording 0 here means they were eligible but got nothing

412. Is anyone in your household eligible to receive a transfer into their widow/pension/Divyang account?

a. Yes

b. No

c. Don’t know

413. How much did you or a household member receive as transfer to the widow/pension/divyang account? (Enter 999 if do not know, 888 refused to respond) 13

414. Do you know of any cash/money that the State government is giving as part of COVID-19 relief?

a. Yes, I am aware of some schemes

b. No, I do not know

415. Since the beginning of the coronavirus crisis and the lockdown, have you or your family members received any OTHER money/DBT from the government into your bank accounts?

a. No, I have not received any OTHER cash transfers.

b. Yes, I have received OTHER cash transfers.

c. I do not know

416. If yes, how much did you receive (in INR) [Enter 999 if dont know, 888 refused to respond] (Do not include PM KISAN/Jan Dhan transfer/pensions)?

417. If you have received cash transfer from the government how did you withdraw the amount?

a. Haven’t received any cash transfer

b. Received but did not try to withdraw

c. Received but was not able to withdraw d. Withdrew by visiting nearest bank branch

e. Withdrew through Bank Mitra/correspondent

f. Through the panchayat office

g. Withdrew through ATM

h. Other, specify

418. Have you benefited from cooked meals being provided by new centers being set up?

a. Yes

b. No

c. Have not tried/did not need to access it

419. Does your household have an MGNREGA job card?

a. Yes, I have a card and I have worked in MGNREGA

b. Yes, I have a card but I have not worked in MGNREGA

c. Applied but no card

d. Not applied

e. Not aware of NREGA

f. Do not know

420. What is your MGNREGA job card number? (999 do not know, 888 refused to respond) 14
621. < If 619 == a> Are there any pending payments that are due to the member from working in MGNREGA, and how much is pending?

a. Yes, (specify amount)

b. Yes, but I don’t know how much

c. No

d. Don't know

421a. How much more you would like to work under MGNREGA this year after lockdown if there won’t be any limit of wage days under MGNREGA?

a. Work the same number of days as last year

b. Work less than last year

c. Work about 1-25 days more than last year

d. Work at least 26-50 days more than last year

422. Are you registered under the BOCW (Board of Building and Other Construction Workers)?

a. Yes

b. No

c. Don’t know

423. Please enter any remarks about the interview or comments from the respondent

500. Economic Impact

500. What kind of economic impact has COVID-19 had on you? (Check all that apply)

- No or little impact
- I lost my job
- I lost my savings
- I cant pay my mortgage anymore
- I cant afford to buy food
- My business is in danger of bankruptcy
- Others specify

501. Have you gone to work in the last month?

- Yes
- No

502. Have you gone to work in the past one week?

- Yes
- No

503. How many people work at your place of work?

- 1-9
- 10-99
- 100+

600. Food Security Impact

600. During the past (30 days), was there a time when, because of lack of money or other resources (FIES Scale):

601. You or others in your household **were worried you would not have enough food to eat** because of lack of money or other resources? (If "Yes", go to question 601a)

- Yes
- No
- Don't Know
- Refused

601a. Was this specifically due to the COVID-19 crisis?

- Yes
- No
- Don't know
- Refused

602. Still thinking about the past 30 days, was there a time when you or others in your household **were unable to eat healthy and nutritious food** because of lack of money or other resources? (If "Yes", go to question 602a)

- Yes
- No
- Don't Know
- Refused

602a. Was this specifically due to the COVID-19 crisis?

- Yes
- No
- Don't know
- Refused

603. Was there a time when you or others in your household **ate only a few kinds of foods** because of lack of money or other resources? (If “Yes”, go to question 603a)

- Yes
- No
- Don’t know
- Refused

603a. Was this specifically due to the COVID-19 crisis?

- Yes
- No
- Don’t know
- Refused

604. Was there a time when you or others in your household **had to skip a meal** because there was not enough money or other resources to get food? (If “Yes”, go to question 604a)

- Yes
- No
- Don’t know
- Refused

604a. Was this specifically due to the COVID-19 crisis?

- Yes
- No
- Don’t know
- Refused

605. Still thinking about the past 30 days, was there a time when you and others in your household **ate less than you thought you should** because of lack of money or other resources? (If “Yes”, go to question 605a)

- Yes
- No
- Don’t know
- Refused

605a. Was this specifically due to the COVID-19 crisis?

- Yes
- No

- Don’t know
- Refused

606. Was there a time when your **household ran out of food** because of lack of money or other resources for food? (If “Yes”, go to question 606a and 606b)

- Yes
- No
- Don’t know
- Refused

606a. How often did this happen?

- Rarely (1 or 2 times)
- Sometimes (3-10 times)
- Often (more than 10 times)
- Don’t know
- Refused

606b. Was this specifically due to the COVID-19 crisis?

- Yes
- No
- Don’t know
- Refused

607. Was there a time when you or others in your household **were hungry but did not eat** because there was not enough money or other resources for food? (If “Yes”, go to question 607a and 607b)

- Yes
- No
- Don’t know
- Refused

607a. How often did this happen?

- Rarely (1 or 2 times)
- Sometimes (3-10 times)

- Often (more than 10 times)
- Don’t know
- Refused

607b. Was this specifically due to the COVID-19 crisis?

- Yes
- No
- Don’t know
- Refused

608. Was there a time when you or others in your household **went without eating for a whole day** because of lack of money or other resources? (If “Yes”, go to question 608a and 608b)

- Yes
- No
- Don’t know
- Refused

608a. How often did this happen?

- Rarely (1 or 2 times)
- Sometimes (3-10 times)
- Often (more than 10 times)
- Don’t know
- Refused

608b. Was this specifically due to the COVID-19 crisis?

- Yes
- No
- Don’t know
- Refused

700. Consumer Behavior Impact

700. Have you refrained from planned larger purchases (in the area of travel for tourism, entertainment, household furnishing, gold jewellery, apparels and so on) due to the outbreak of the coronavirus?

- Yes

- No

701. During the early stages of the outbreak of the coronavirus, have you started to store larger quantities of food supplies (panic buying) at home than before?

-

S.no.	Food group	Yes	No
1	Cereals and their products (rice, wheat, maize, sorghum, millets and their products such rice flakes, corn flakes, noodles and so on)		
2	Roots, tubers, plantains and products (potato, sweet potato, cassava, yam, plantain and other related products)		
3	Pulses, seeds and nuts and their products (pulses such redgram, green gram, black gram, Bengal gram and its products; soybean and soy-based prducts such as tofu; nuts such as coconut, walnut, groundnut and their products such as oils, peanut butter and so on)		
4	Milk and milk products (fresh cow milk, buffalo milk, goat milk, camel milk, flavored milk and other products such as fermented milk, ghee, butter, buttermilk, cheese, cream, sour cream and so on)		
5	Eggs and their products (hen, duck, geese, quail, turkey eggs both fresh and processed)		
6	Fish, shell fish and their products (freshwater fish, processed fish, marine fish, shell fish and processed products such as fish		

	sauce, fish oil, canned fish, dried fish and so on)		
7	Meat and meat products<br>(offal, mammals, birds meat and its products such as marinated meat, canned, tinned meat, sausages, dried meat and so on)		
8	Insect, grubs and their products<br>(all terrestrial insects such as spiders, mites, ticks, beetles; grubs such as earthworms and so on)		
9	Vegetables and their products<br>(leafy vegetables, yellow and orange vegetables, bulb vegetables, and all other vegetables and their products such as vegetable pastes, pickled vegetables and so on)		
10	Fruits and their products<br>(fresh fruits such as orange, apple, grapes and processed fruits such as canned fruit, sun-dried fruits and so on)		
11	Fats and oils<br>(all vegetable fats and oils and animal fats)		
12	Sweets and sugars<br>(dough-based sweets, chocolate-based sweets, fruit and nut-based sweets, dairy-based sweets, jellies, sweet bars and sugars, honey and others)		
13	Spices and condiments<br>(cloves, cardamom, cinnamon and so on)		
14	Beverages<br>(alcoholic drinks, drinking water, soft drinks, fruit concentrates, extracts, fruit juices, vegetable juices and so on)		
15	Foods for particular nutritional uses		

	(infant formulas, ready-to-eat meals for young children, foods for weight reduction, foods for sports people)		
16	Food supplements<br>(protein supplements, herbal supplements, probiotic foods and so on)		
17	Food additives<br>(sweeteners, flavorings, colorants, yeast and so on)		
18	Composite dishes<br>(rice-based, vegetable-based, meat-based, fruit-based, soups and so on)		
19	Savoury snacks<br>(includes crisps and curls)		
Note: The detailed food groups have been extracted from the FAO/WHO GIFT food group classification. http://www.fao.org/gift-individual-food-consumption/methodology/food-groups-and-sub-groups/en/			

-

702. Since the outbreak of the coronavirus, have you started to store larger quantities of medical supplies at home than before?

- Yes
- No

703. How many years do you think the coronavirus outbreak will last?

- 1 year
- 2 years
- 3 years
- More than 3 years
